# Outcomes of Kidney Transplantation in Patients with Autosomal Dominant Polycystic Kidney Disease: Our Experience Based on 35-Years Follow-Up

**DOI:** 10.3390/diagnostics12051174

**Published:** 2022-05-08

**Authors:** Tsung-Yin Tsai, Cheng-Hsu Chen, Ming-Ju Wu, Shang-Feng Tsai

**Affiliations:** 1 Division of Nephrology, Department of Internal Medicine, Taichung Veterans General Hospital, Taichung 40705, Taiwan; g0955102226@gmail.com (T.-Y.T.); cschen920@vghtc.gov.tw (C.-H.C.); wmj530@vghtc.gov.tw (M.-J.W.); 2 Department of Life Science, Tunghai University, Taichung 40704, Taiwan; 3 Department of Post-Baccalaureate Medicine, College of Medicine, National Chung Hsing University, Taichung 40227, Taiwan; 4 School of Medicine, National Yang-Ming University, Taipei 11265, Taiwan

**Keywords:** autosomal dominant polycystic kidney disease, renal transplantation, patient survival, malignancy, new onset diabetes mellitus after transplantation

## Abstract

Background and objectives: For patients with end-stage renal disease (ESRD), the best replacement therapy is renal transplant (RTx) to ensure life with good quality. Autosomal dominant polycystic kidney disease (ADPKD) is a genetic disorder and a common cause of ESRD. Different from ESRD of other causes, ADPKD patients need careful pre-RTx evaluations like detecting the presence of intracranial aneurisms, cardiac manifestations, and complications of liver and renal cysts. Materials: We retrieved a total of 1327 RTx patients receiving 1382 times RTx (two recipients with three times, 48 recipients with two times) over the last 35 years. Only 41 of these patients were diagnosed with ADPKD. Results: At the first RTx, patients’ ages were 42.9 ± 12.6 (mean ± SD) years. Ages of the ADPKD group (52.5 ± 10.1 years) were older than the non-ADPKD group (42.7 ± 12.7 years, *p* = 0.001). We found more cell mediated and antibody mediated rejection (29.3% vs. 26.0%, and 22.0% vs. 7.0%; both *p* < 0.001), new onset diabetes after transplant (NODAT) (21, 51.2% vs. 326, 25.3%; *p* = 0.005), and worse graft survival (*p* < 0.001) in the ADPKD group, and with the development of more malignancies (18; 43.9% vs. 360; 28.0%; *p* = 0.041). The long-term patient survivals were poorer in the ADPKD group (38.9% vs. 70.3%; *p* = 0.018). ADPKD was found as an independent risk factor for long-term patient survival (HR = 2.64, 95% CI 1.03–6.76, *p* = 0.04). Conclusions: Patients with ADPKD-related ESRD developed more NODAT, and also more malignancies if not aggressively surveyed before surgery. Due to poor long-term graft and patient survivals, regular careful examinations for NODAT and malignancies, even in the absence of related symptoms and signs, are highly recommended in the follow-ups.

## 1. Introduction

Renal transplantation (RTx) is the best renal replacement therapy for patients with end-stage renal disease (ESRD). The outcome of RTx is better than that of hemodialysis, even when considering the long-term outcome of recipients with autosomal dominant polycystic kidney disease (ADPKD) as compared with the non-ADPKD group [1]. In a 2009 review [2], complications after RTx were found to be no worse than in the general population, and complications directly related to ADPKD are rare. Therefore, RTx is still recommended for patients with ADPKD-related ESRD. However, long-term outcomes vary across kidney transplants to recipients of different renal diseases. Prior to dialysis, any inherited systemic disorder is already rather complex, and that is further complicated after RTx. Related to patient survival [3], a number of factors are involved, like those with donors (e.g., quality of allograft kidney) and recipients. As for recipients, the different time-related conditions: (a) before RTx (e.g., baseline ADPKD status, quality of care for chronic kidney disease (CKD), the initial choice of hemodialysis or peritoneal dialysis), (b) during RTx (e.g., risk of operation due to cysts burden, thromboembolism, and ischemic time), and (c) after RTx (status of mismatch, rejection, infection, metabolic syndrome and cancer) are all together associated with long-term survival. In this study, we focused on the long-term outcomes i.e., (a) patient survival, of recipients of ADPKD after transplantation (NODAT), and (b) new cancers.

The patient survival outcome of ADPKD patients receiving RTx is inconsistent across previous reports in that it is either (a) similar to other cause-related ESRD [4], (b) better than the non-ADPKD group [1], or (c) worse than the non-ADPKD group [5]. The discrepancy in results could be due to relatively short follow-up periods, and old publications (since new immunosuppressants are used more often in the recent era). Furthermore, malignancy and NODAT both influence survival. As for the long-term risk for malignancy in ADPKD-related recipients, it remains a possibility. Cancers were reported to associate with ADPKD [6]. In 1934, Walters found more renal carcinoma in ADPKD patients [7]. According a nationwide study in Taiwan, ADPKD is also associated with more cancers (e.g., liver, colon and kidney) [8]. But such higher risks for malignancy were not found in another 2010 study [9]. The major cause of death for ESRD is well-known to be cardiovascular disease (CVD), regardless it is the case of CKD (not yet under dialysis) [10] ESRD (under dialysis) [11], or even after RTx [12]. Thus, NODAT is also an important factor for long-term survival of patients. Here, we conducted this long-term follow-up study to determine the patient survival rate of ADPKD recipients, and compared results with the non-ADPKD patients.

## 2. Materials and Methods

### 2.1. Study Population

This respective cohort study was aimed to evaluate the long-term outcomes of recipient patients in our hospital, including their patient survival, malignancy and NODAT. Our hospital, the Taichung Veterans General Hospital, is a medical center in central Taiwan. In the past 35 years, we performed RTx on 1327 patients (or 1382 operations). Their medical records were first reviewed, and their clinical data were then analyzed. In our protocol of RTx, our usual induction therapy (one day before RTx) included methylprednisone 500 mg, tacrolimus 0.2 mg/kg/day or cyclosporine 9 mg/kg/day, cellcept or myfortic (four tablets bid if body weight > 50 kg or three tablets bid if body weight < 50 kg). Our study was approved by Ethics Committee of Taichung Veterans General Hospital, IRB approval CE18192. All methods were carried out in accordance with relevant guidelines and regulations and informed consent was obtained from each subject.

### 2.2. Data Collection

All data of this cohort were obtained from medical records. At the time of renal transplantation, we took the patient’s baseline data such as gender, age, body height (cm), body weight (kg), systolic or diastolic blood pressure (SBP and DBP), and the duration of follow-up. Blood urea nitrogen (BUN) (mg/dL), and serum creatinine (mg/dL) were collected at least a month after RTx under stable condition. Other blood sample data were collected for the following parameters: white blood cell (WBC) (/mm^3^), hemoglobin (g/dL), neutral and lymphocyte ratio (%), platelet count (/mm^3^), uric acid (mg/dL), potassium (meq/L), calcium (mg/dL), phosphate (mg/dL), serum albumin (g/dL), total protein (g/dL), aspartate transaminase (AST) (U/L), alanine transaminase(ALT) (U/L), total cholesterol (mg/dL), triglyceride (mg/dL), low-density lipoprotein (LDL) (mg/dL), high-density lipoprotein (HDL) (mg/dL), fasting and postprandial blood sugar (mg/dL), glycated hemoglobin (%), and γ-glutamyl transpeptidase (U/L). Data from urinary tests were collected for urinary protein-creatine (g/g creatinine), and urinary albumin-creatinine ratio (mg/g creatinine). For tumor markers reportedly associated with ADPKD-related cancers, we collected the alpha-fetoprotein (ng/mL), carcinoembryonic antigen (ng/mL), carbohydrate antigen 199 (U/mL) [13], and cancer antigen 125 (U/L).

### 2.3. Diagnosis of Diabetes Mellitus (DM), NODAT and ADPKD

DM was diagnosed according to the criteria of American Diabetes Association [14,15]. Based on a 2003 consensus on treating NODAT, HbA1c is not recommended to be used for assessment within the first three months after transplantation [16]. The reason is that during this early postoperative period, renal function has not fully recovered, and HbA1c may underestimate the control of sugar levels. The diagnosis of ADPKD is as follows. If family history is positive, the age-dependent ultrasonographic criteria are used for the diagnosis according to Ravine et al. [17,18]. In the absence of family history, we made the ADPKD diagnosis according to Gabowl [19]. No genetic test was used for diagnosis in this study, since in Taiwan it is the practice not to use genetic tests for this confirmation in the application for catastrophic illness cards. We also searched the database of National Health Insurance Administration Ministry of Health and Welfare, the ICD code (Q61.3 and Q61.2 from ICD10; 753.12 and 753.13 from ICD9), to check if those patients owning catastrophic illness cards were recorded with the diagnosis of ADPKD. Polycystic occurrences after RTx were considered as acquired renal cysts and not ADPKD.

### 2.4. Outcome Assessment

Mortality was confirmed in the medical records and in the case of deaths we also confirmed withdrawal of these patients from the National Health Insurance scheme. The diagnosis of various malignancies was also confirmed in the medical records and we also cross-checked them in the database of National Health Insurance Administration of the Ministry of Health and Welfare. In Taiwan, once diagnosed with a malignancy, patients would normally apply for the catastrophic illness cards. The National Health Insurance scheme is a very comprehensive public healthcare system, covering >99% of the island’s 23 million people [20]. The patient survivals were plotted according to ADPKD or not. Furthermore, cyclosporine (Novartis/Germany) has been used in Taiwan since 1985 and tacrolimus (Astellas/Ireland) has been used in Taiwan since 1998. We further separated patient survival curves in ADPKD and non-ADPKD groups into three time periods: before 1985, 1985–1998 and after 1998. As for the periods of mammalian target of rapamycin inhibitor, it was never used in our institute in in the beginning of RTx. Cell-mediated rejection was collected according the post-RTx methylprednisolone. Antibody-mediated rejection was confirmed if recipients received Rituximab, Bortezomib, Human Immunoglobulin, or therapeutic plasmapheresis. Until follow-up of this study, we collected the data of graft failure, including returning to hemodialysis or peritoneal dialysis.

### 2.5. Statistical Methods

Data were expressed as the mean ± SD for continuous variables, or as numbers (percentages) for categorical variables. The Mann–Whitney U test was used for comparing continuous variables, and the Chi-square test for categorical variables. The Kaplan-Meier curve was plotted to depict patient survival. The Cox proportional hazard regression model (univariable and multivariable) was used to extract factors affecting renal survival. Statistical significant differences were set at *p* < 0.05. All statistical procedures were performed using the SPSS statistical software package, version 17.0 (Chicago, IL, USA).

## 3. Results

**Figure 1:**
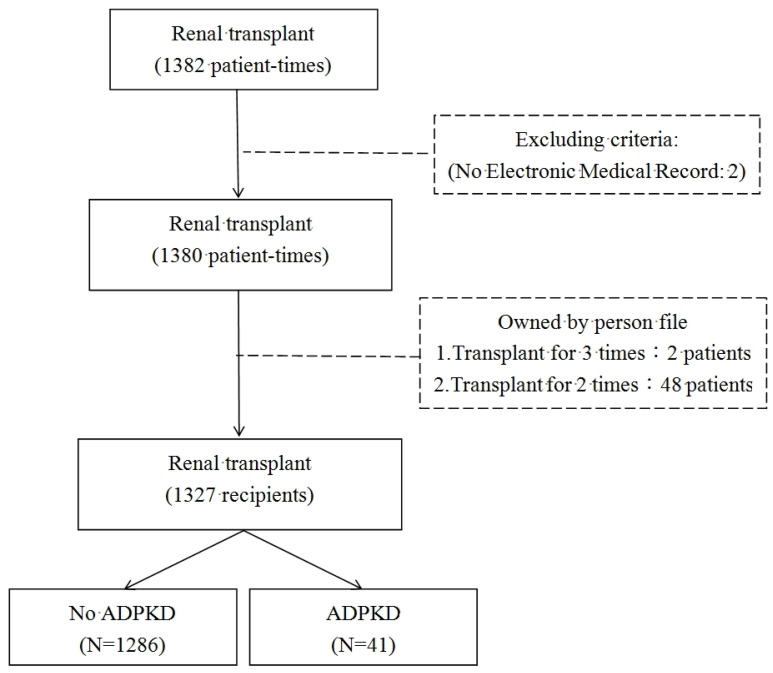
Flowchart of the renal transplant recipients for the ADPKD group and non-ADPKD group.

We first examined 1382 recipients of RTx. After exclusion, 1327 patients remained to be analyzed (Figure 1). Baseline characteristics are summarized in Table 1. Most of them were non-ADPKD related ESRD patients (n = 1286) and very few of them were ADPKD-related ESRD (n = 41). In all 41-ADPKD patients receiving RTx, 21 patients had liver involvement, and no one had pancreatic involvement. No hepatitis B or C was noticed in these 41 patients. No one received pre-RTx nephrectomy in this cohort and no one received a liver-kidney transplant simultaneously.

This cohort while receiving RTx was relatively young (42.9 ± 12.6 y/o), and they had already undergone regular follow-up for an average of 11.6 years. Recipients had mild hypertension (144.4 ± 22.6 mmHg of SBP) and good renal functions (1.3 ± 0.4 mg/dL of SCr, 256.8 ± 780.9 mg/g of urinary ACR). During the enrollment period, recipients had relatively stable levels of serum electrolytes (potassium, calcium and phosphate) and normal liver function (23.0 ± 16.5 U/L of AST, and 25.3 ± 25.2 U/L of ALT). Hyperglycemia (135.8 ± 71.0 of fasting blood glucose) was noticed after >10 years of follow-up. Of them, 26% recipients had NODAT, and 7% of recipients died. Cancers were detected in 28.5% of recipients. Compared with the non-ADPKD group, the ADPKD-group was older (48.6 ± 10.8 vs. 42.7 ± 12.7, *p* = 0.005), with more patients receiving hemodialysis before RTx (88.9% vs. 47.8%, *p* = 0.026), and they had a higher mortality (19.5% vs. 6.6%, *p* = 0.006), more NODAT (46.3 vs. 25.3%, *p* = 0.005), more cell mediated and antibody mediated rejection (29.3% vs. 26.0%, and 22.0% vs. 7.0%; both *p* < 0.001), and more malignancies (43.9% vs. 28.0%, *p* = 0.041). More ADPKD recipients returned to hemodialysis than non-ADPKD recipients (29.3% vs. 20.3%, *p* < 0.001).

**Figure 2:**
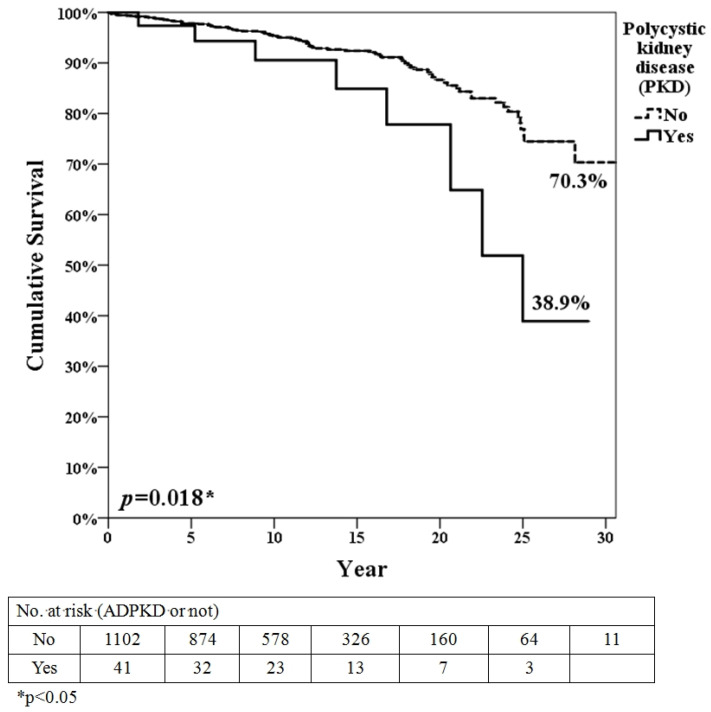
Patient survivals of RTx due to underlying ADPKD or not.

For patients’ long-term survival (Figure 2), after an average of 11.6 years of follow-up, the ADPKD-group was poorer compared with the non-ADPKD group (38.9% vs. 70.3%, *p* = 0.018). Because only three RTx which was performed before cyclosporin before 1985 (before cyclosporine), we separate patients survival as before 1998 (after cyclosporine and before tracrolimus) (Supplementary Figure S1) and after 1998 (after tacroliumus) (Supplementary Figure S2). ADPKD groups showed the trend of worst patient survival compared to the non-ADPKD group in both time periods. The analysis of the cause of death using a Cox regression model is shown in Table 2. In the univariate analysis, older ages (HR = 1.10, 95% CI = 1.08–1.12), DM (HR = 1.88, 95% CI = 1.25–2.83), polycystic kidney disease (HR = 2.34, 95% CI = 1.13–4.83) and ADPKD (HR = 5.73, 95% CI = 2.29–14.35) were risk factors for long-term mortality. In the multivariate analysis, aging is the only risk factor for mortality in model 1 (HR = 1.09, 95% CI = 1.07–1.12). But in multivariate analysis in model 2, aging (HR = 1.09, 95% CI = 1.07–1.11) and ADPKD (HR = 2.64, 95% CI = 1.03–6.76) are both independent risk factors for mortality. Our finding was not consistent with a previous study in which ADPKD is not a risk factor for the occurrence of solid cancers after transplantation (HR 0.96; *p* = 0.89). In addition, the major cause of death for ESRD is CVD, from CKD (not yet under dialysis) [10] to ESRD (under dialysis) [11], even after renal transplantation [12].

The cause of patient mortality in both groups are shown in Supplementary Table S1. Most deaths in both groups were attributable to cardiovascular disease (50% in ADPKD group and 47.1% in non-ADPKD group). In the ADPKD group, other recipients died due to cancer (25%) and infection (12%). In the non-ADPKD group, other recipients died due to infection (26%) and cancer (15.3%). Detailed information of types of malignancy were presented in Supplementary Table S2. In the ADPKD group, most recipients had colon cancer, lung, urinary bladder, and renal cancers. In the non-ADPKD group, most recipients had colon and liver cancer.

## 4. Discussion

The comparison of patient survival rates after RTx between ADPKD and non-ADPKD patients has reached no consensus until now. The first study on this issue was in 1990 [21] and had the following finding: the overall patient survivals and patients surviving with a functioning first renal allograft are similar between the ADPKD and non-ADPKD groups. In another study in 1994 [22], similar overall survivals appeared between the two groups; however, with a worse age-adjusted relative risk of 2.07 (95% confidence interval [95% CI]: 1.12–3.80) for CV events and CV related mortality. Similar overall patient survival rates were confirmed in the later five studies [23,24,25,26,27]. In all previous studies (i.e., all done before 2002) [21,22,23,24,25,26,27], case numbers were small with relatively short durations of follow-up (<10 years). In 2005, Johnston et al., reported that, based on the analysis of a multifactorial model, survival rates of ADPKD patients were better (at 5% levels, *p* = 0.036) compared to other causes of ESRD [1]. In 2008, Roozbeh et al., also showed that patient outcomes (after short- and long-term follow-up) were better in the ADPKD group than in the controls [4]. In 2011, a nationwide longitudinal study showed that ADPKD patients (n = 534), when compared with non-ADPKD patients (n = 4,779), have better graft survivals, more thromboembolic complications, more metabolic complications, and increased incidence of hypertension [28]. In summary, the last three studies supported better patient survivals as published within the period from 2005 to 2011 [1,4,28]. Illesy et al., reported to the contrary that the one-, three-, and five-year overall patient survival rates in ADPKD recipients weas 77.5%, 70.0%, and 62.5%, versus 86.5%, 79.8%, and 73.4% in the non-ADPKD patients (*p* = 0.013), respectively [5]. That study showed poorer survivals in the ADPKID group compared to the non-ADPKD group (*p* = 0.018), a finding that is consistent with ours. In our multivariate Cox regression model, ADPKD was the independent risk factor for long-term patient survival (HR = 2.64, 95%CI = 1.03–6.76, *p* = 0.044; after adjustments were made for age, sex, and DM).

A number of factors could account for the worse patient survivals found in this study. First, we had more cases (41 ADPKD vs. 1286 non-ADPKD patients) compared with others, and consequently smaller differences were detected. Second, we had the longest follow-up duration (mean duration of follow-up is 11.6 years, while the longest was up to 35 years) compared with others. Therefore, regarding long-term patient survival, we were able to detect factors (e.g., NODAT, post-RTx malignancy) that required longer times to show their effects. Third, regarding other studies on similar patient survival rates, their conclusions are different according to the different time periods in which the studies were conducted: 1990–2002 (seven studies: similar) [21,22,23,24,25,26,27], 2005–2011 (three studies: better) [1,4,28] and 2017–2019 (two studies, including this study: worse) [5]. With newer immunosuppressive protocols, improved surgical techniques and transplantation in older and sicker patients, the outcomes could also change accordingly. On top of these are newer medications for the treatment of CVD, NODAT and malignancy. Therefore, risks and determinants of mortality in the recipients of RTx need to be reassessed, which was consistent with our study (more rejection in ADPKD groups). In our study, we found more NODAT in the ADPKD group (46.3% vs. 25.3%, *p* = 0.005). The more NODAT could cause more CVD and CVD-related mortality after longer follow-ups. Fourth, before RTx, we had more patients receiving hemodialysis in the ADPKD group. Patients with ESRD choosing hemodialysis as the treatment modality developed more comorbidities [29,30], including prior histories of myocardial infarction (*p* = 0.031), DM (*p* = 0.001) and congestive heart failure (*p* = 0.003) [29]. The different baseline conditions also affect the long-term patient outcomes. Fifth, the poorer outcome in the ADPKD group is due to older ages (42.7 vs. 48.6 years old, *p* = 0.005). Age itself is a risk factor for long-term mortality. Also, PADKD (including cyst progression and extra-renal manifestation) progresses more aggressively in older patients. The progression of cysts induces more cyst-related complications (e.g., infection, cyst compression, and rupture) as well as more non-cyst related complications (e.g., intracranial aneurism and cardiac manifestation). Complications of ADPKD increase with aging, and as a result could shorten patient survivals after RTx. Finally, over the various study periods in the literature, no consensus has been reached regarding the standard pre-RTx preparations for ADPKD patients (e.g., pre-RTx nephrectomy, pre-RTx antibiotics, and a detailed malignancy survey). No consensus was reached about this, even though native nephrectomy for ADPKD was considered to be safely performed in a case of refractory symptoms [31].

Factors inducing cancers are complex. ADPKD has been considered as a tumor-like disease [6]. Therefore, an aggressive tumor survey should be performed before RTx. Originally, cancer incidence was thought to be lower after RTx because patients with cancers and high risks for malignancy were typically excluded for surgery. But with sufficiently long periods of follow-up, and with greater exposures to immunosuppressive drugs, the incidence of malignancy is still higher [32]. However, even with biological association [6], clinical evidence remained weak between ADPKD (not yet RTx) and malignancy until a nationwide study was published in 2016 [8]. In that study [8], ADPKD without ESRD was shown to be a risk factor for cancers of the liver, colon, and kidney, but results are not supported by laboratory data [33]. As for ADPKD-related ESRD, it is not a risk factor for the occurrence of solid cancers after transplantation (HR = 0.96; *p* = 0.89) [9]. Similarly, the prevalence of cutaneous malignancies (7.9% vs. 7.4%, *p* = 0.71) and kidney cancers (0.4% vs. 0.6%, *p* = 0.50) are shown to be similar between the two groups [28]. In another 2014 study on 10,166 kidney recipients [34], the unadjusted incidence of cancer is higher than the general population (IRR = 1.10; 95% CI = 1.01–1.20). But after adjusting for age, sex, ethnicity, dialysis duration, and time since RTx, the cancer incidence becomes lower instead (IRR = 0.84; 95% CI = 0.77–0.91). In summary, the increased incidence of malignancy in ADPKD (not yet ESRD or RTx) [33] is not found after RTx [9,28,34]. That is reasonable, because ADPKD patients with pre-existing cancers or at high risk for cancers are contraindicated for RTx. In other words, the baseline condition of ADPKD patients receiving RTx is biased towards those without malignancy or with little risks for malignancy. However, the real association between ADPKD after RTx and malignancy in Taiwan is unknown. In this study, pre-RTX cancer prevalence in non-ADPKD and ADPKD were comparable (even though more cancers seemed to appear in the ADPKD group, 9.8% vs. 4.1%, *p* = 0.095). After RTx the cancer prevalence in both groups increased in long-term follow-ups. The higher cancer prevalence after RTx is not surprising due to the aging process and immunosuppressant medication. However, we found higher cancer prevalence in the ADPKD group (24.3% vs. 19.8%), a new finding that is reported here for the first time [9,28,34]. The discrepancy in our results with the literature could be due to the following. First, we had longer duration of follow-ups, and we used more new immunosuppressants (e.g., tacrolimus, which could impose higher risks for cancers) [35]. Second, we used fewer rapamycin (mTOR) inhibitors, which have potential advantages in virus-associated posttransplant malignancies as well as anti-cancer properties [36]. Third, we may not have screened malignancies before RTx as aggressively as others did. The higher post-RTx malignancy in our ADPKD group also predisposed patients to higher long-term mortality.

Before RTx, the baseline prevalence of DM were similar between the two groups (*p* = 0.537). After long-term follow-up, their DM prevalence increased, especially in the ADPKD group (an increment of 41.4%). After RTx, the DM prevalence was higher in the ADPKD group (*p* = 0.005). In other words, NODAT appeared more significant in the ADPKD group. This finding is consistent with other studies [37,38]. While the exact mechanism remains elusive, pancreatic and hepatic factors related to insulin resistance genes co-transmitted with PKD1 and PKD2 mutations might affect insulin secretion and gluconeogenesis. More NODAT in the ADPKD group could also have rendered these patients more susceptible to CVD and CVD-related mortalities.

There are several limitations in this study. First, we did not have data on maintenance immunosuppressants. However, we collected data for methylprednisolone pulse therapy. Second, we did not have information about quality of life in this cohort. We will investigate this part in our future study. Finally, a 35-year study brings together patients from different generations and different treatment schedules across a long period of time. Furthermore, different physicians prescribed different amounts of prednisone, which had different pressure for NODAT, and different amounts of immunosuppressants, which caused pressure for cancer.

## 5. Conclusions

Patients with ADPKD-related ESRD should be more carefully followed up after RTx. The long-term patient and graft survivals may be not as good as ESRD due to other causes. Patients experienced worse long-term patient survivals due to more rejection, followed by more NODAT, worse graft survival and more malignancy (particularly if cancer survey before RTx was not performed thoroughly). Therefore, we recommend regular examinations for NODAT and malignancy in these patients even in the absence of any symptoms and signs.

## Figures and Tables

**Table 1:** Baseline characteristics of renal transplantation recipients according to ADPKD or not ADPKD.

	Without ADPKD (n = 1286)		With ADPKD (n = 41)		Total (n = 1327)		*p* Value
Age (yrs) (Mean ± SD)	42.7 ± 12.7		48.6 ± 10.8		42.9 ± 12.6		0.005
Male gender	713 (55.4%)		28 (68.3%)		741 (55.8%)		0.141
Vintage of RTx (yrs)	10.6 (5.8–16.4)		12.0 (5.4–18.2)		10.6 (5.8–16.5)		0.740
Duration of follow-up (yrs)	11.6 ± 7.4		12.0 ± 7.7		11.6 ± 7.4		0.802
Systolic blood pressure (mmHg)	144.3 ± 15.3		145.3 ± 10.2		144.4 ± 22.6		0.903
Diastolic blood pressure (mmHg)	85.3 ± 10.2		88.6 ± 11.3		85.4 ± 15.2		0.123
Body height (cm)	163.8 ± 7.2		162.2 ± 7.3		162.9 ± 8.3		0.832
Body weight (kg)	62.3 ± 10.3		63.3 ± 11.3		62.2 ± 12.6		0.863
Body mass index (kg/m^2^)	23.3 ± 4.0		22.3 ± 3.9		23.4 ± 3.8		0.993
Blood urea nitrogen (mg/dL)	52.9 ± 23.2		53.8 ± 22.5		52.5 ± 26.8		0.863
Serum creatinine (mg/dL)	1.29 ± 0.6		1.30 ± 0.3		1.3 ± 0.4		0.756
White blood cell (/μL)	11069 ± 4063		11630 ± 6530		11016.2 ± 4759.3		0.756
Hemoglobin (g/dL)	10.4 ±3.0		10.3 ± 1.1		10.3 ± 2.3		0.856
Neutrophil (%)	84.3 ± 11.0		83.9 ± 9.3		84.5 ± 11.6		0.746
Platelet (×10^3^/μL)	197.3 ± 69.3		196.3 ± 66.3		196.5 ± 73.7		0.456
Uric acid (mg/dL)	6.3 ± 3.0		6.1 ± 1.9		6.2 ± 2.1		0.896
K (meq/L)	4.4 ± 0.2		4.3 ± 0.2		4.4 ± 0.8		0.846
Ca (mg/dL)	8.3 ± 2.0		8.1 ± 0.9		8.1 ± 1.5		0.635
P (mg/dL)	4.4 ±1.0		4.2 ± 0.9		4.3 ± 1.9		0.456
Blood albumin (g/dL)	3.9 ± 0.7		3.8 ± 0.8		3.9 ± 0.6		0.674
Total protein (g/dL)	6.5 ± 0.8		6.4 ± 1.5		6.4 ± 1.0		0.456
Aspartate transaminase (U/L)	24 ± 12.6		22.9 ± 10.3		23.0 ± 16.5		0.697
Alanine transaminase (U/L)	23.8 ±24		24.9 ± 15.3		25.3 ± 28.2		0.623
Total cholesterol (mg/dL)	190.8 ± 63.3		188 ± 42.2		189.7 ± 52.6		0.296
Triglyceride (mg/dL)	149 ± 80		135 ± 68		138.7 ± 100.0		0.753
Low-density lipoprotein (mg/dL)	110.9 ± 50.3		112 ± 33.9		111.7 ± 42.1		0.2698
High-density lipoprotein (mg/dL)	53.7 ± 20.3		51.9 ± 22.3		52.0 ± 17.5		0.963
Blood glucose (mg/dL)	135.9 ± 60.2		134.8 ± 70.2		135.8 ± 71.0		0.756
Glycated hemoglobin (%)	6.3 ± 2.0		6.1 ± 2.3		6.1 ± 1.3		0.369
Urinary protein-creatinine ratio (g/g creatinine)	0.59 ± 2.0		0.62 ± 2.3		0.6 ± 1.7		0.458
Urinary albumin-creatinine ratio (mg/g creatinine)	254.9 ± 300.2		255 ± 720		256.8 ± 780.9		0.692
Alkaline phosphatase (U/L)	88.2 ± 56.3		89.3 ± 77.2		89.1 ± 65.4		0.752
Direct bilirubin (mg/dL)	0.1 ± 0.09		0.1 ± 0.03		0.1 ± 0.1		0.647
Total bilirubin (mg/dL)	0.4 ± 0.18		0.4 ± 0.2		0.4 ± 0.2		0.745
γ-Glutamyl transpeptidase (U/L)	95.08 ± 89.2		94.0 ± 85		94.9 ± 110.9		0.852
Alpha-fetoprotein (ng/mL)	4.9 ± 3.8		4.9 ± 4.0		4.9 ± 4.1		0.951
Carcinoembryonic antigen (ng/mL)	1.98 ± 0.98		2.0 ± 0.74		2.0 ± 1.0		0.364
Carbohydrate antigen 199 (U/mL)	13.8 ± 9.8		13.1 ± 8.3		13.2 ± 11.9		0.458
Cancer antigen 125 (U/mL)	35.9 ± 35		33.8 ± 23		33.9 ± 40.0		0.856
Death ^f^	85	(6.6%)	8	(19.5%)	93	(7.0%)	0.006
Diabetes mellitus	439	(34.1%)	21	(51.2%)	460	(34.7%)	0.036
Before RTx ^f^	113	(8.8%)	2	(4.9%)	115	(8.7%)	0.573
After RTx	326	(25.3%)	19	(46.3%)	345	(26.0%)	0.005
Onset after RTX (days)	986 ± 835.2		1100 ± 9302		1088.5 ± 1069.1		0.5866
Cancer	360	(28.0%)	18	(43.9%)	378	(28.5%)	0.041
Before RTx ^f^	53	(4.1%)	4	(9.8%)	57	(4.3%)	0.095
After RTx	307	(23.9%)	14	(34.1%)	321	(24.2%)	0.184
Cell-mediated rejection	335	26.0%	12	(29.3%)	347	26.1%	<0.001
Antibody-mediated rejection	99	7.0%	9	(22.0%)	99	7.5%	<0.001
Hemodialysis	598	(47.8%)	40	(88.9%)	638	(47.6%)	0.026
Peritoneal dialysis	155	(11.6%)	5	(11.1%)	160	(11.9%)	0.805
Hematuria	10	(0.78%)	15	(36.6%)	25	(1.9%)	0.045
Flank pain	15	(0.66%)	10	(24.4%)	25	(1.9%)	0.036
Fullness sensation	20	1.6%	3	7.3%	23	1.7%	0.563
Return to hemodialysis	271	20.3%	12	29.3%	283	21.3%	<0.001

Mann-Whitney U test. Chi-Square test. ^f^ Fisher′s Exact test. ADPKD: autosomal dominant polycystic kidney disease; RTx: renal transplantation.

**Table 2:** Univariate and multivariate Cox regression models for the causes of death in RTx.

	Univariate Analysis			Multivariate Analysis (Model 1)			Multivariate Analysis (Model 2)		
	HR	(95% CI)	*p* Value	HR	95% CI	*p* Value	HR	95% CI	*p* Value
Age	1.10	(1.08–1.12)	<0.001 **	1.09	(1.07–1.12)	<0.001 **	1.09	(1.07–1.11)	<0.001 **
Sex (M vs. F)	1.05	(0.70–1.58)	0.818	1.06	(0.70–1.60)	0.796	1.05	(0.69–1.59)	0.830
DM	1.88	(1.25–2.83)	0.003 **	1.24	(0.81–1.89)	0.314	1.27	(0.83–1.94)	0.263
Polycystic kidney disease (PKD)	2.34	(1.13–4.83)	0.022 *	1.75	(0.84–3.65)	0.137			
Group									
No ADPKD	reference						reference		
Polycystic after RTX	1.17	(0.37–3.72)	0.788				1.13	(0.35–3.62)	0.838
Polycystic before RTX	5.73	(2.29–14.35)	<0.001 **				2.64	(1.03–6.76)	0.044 *

Cox regression. * *p* < 0.05, ** *p* < 0.01.

## Data Availability

Not applicable.

## Supplementary Materials

The following supporting information can be downloaded at: https://www.mdpi.com/article/10.3390/diagnostics12051174/s1, Figure S1: Overall patients survival before 1998 (after cyclosporin and before Tacrolimus); Figure S2: Overall patient survival after 1998 (after Tacrolimus); Table S1: Cause of death; Table S2: Detailed information of types of malignancy.
