# Supplementary material for: Outcomes of Kidney Transplantation in Patients with Autosomal Dominant Polycystic Kidney Disease: Our Experience Based on 35-Years Follow-Up
Source: Diagnostics (Basel). 2022 May 8;12(5):1174. doi: 10.3390/diagnostics12051174 (PMC9139921; doi:10.3390/diagnostics12051174)
Supplement: Supplementary file 1 [file diagnostics-12-01174-s001.zip › diagnostics-1679824-supplementary.pdf]

Supplementary data

Figure S1. Overall patients survival before 1998 (after cyclosporin and before Tacrolimus)

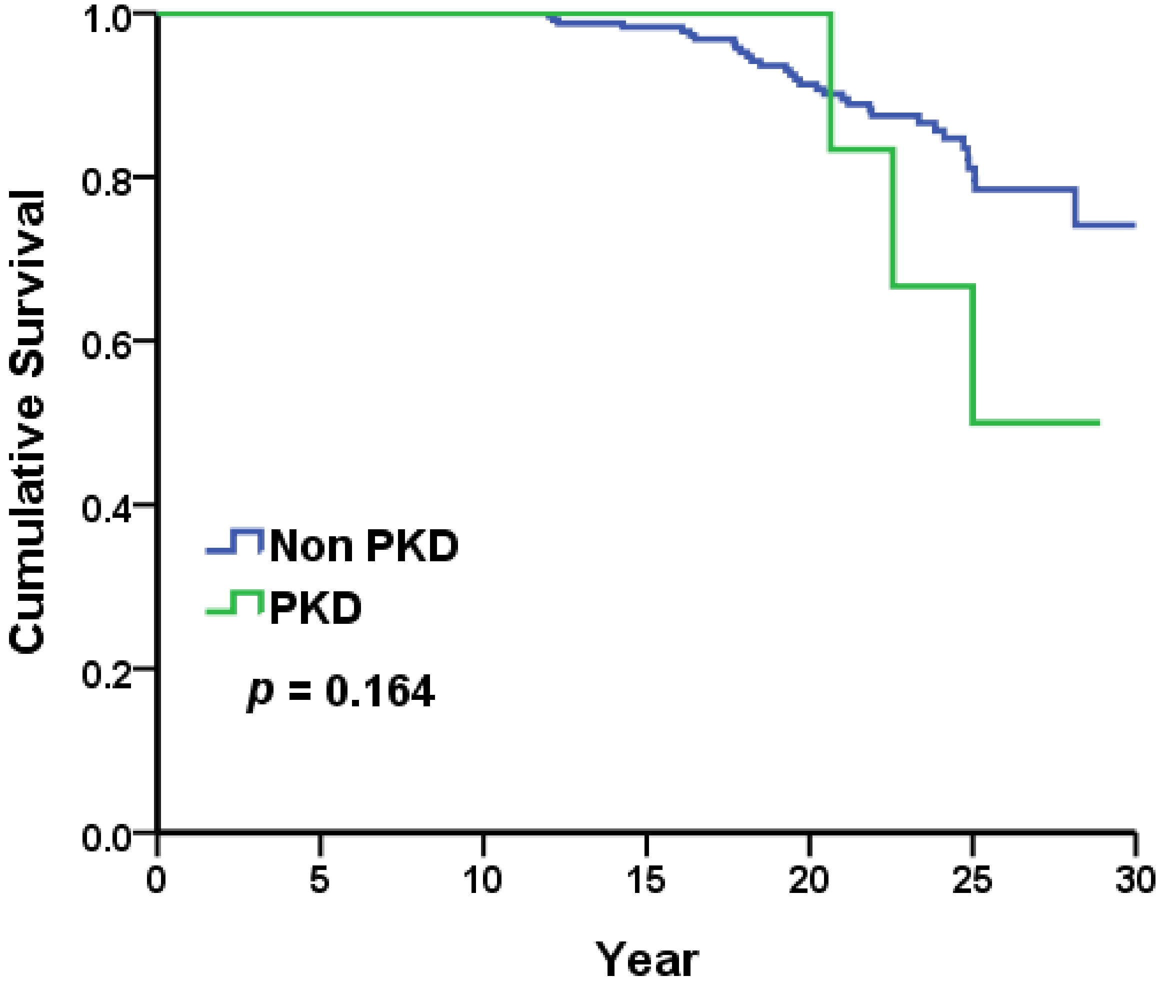

Overall survival (before 1998)

	Total	Death	Censored		Survival rate (%)						p for log rank
			n	%	5yr	10yr	15yr	20yr	25yr	30yr	
PKD											
No	299	33	266	89.0%	100.0%	100.0%	98.3%	91.3%	81.0%	74.1%	0.164
Yes	8	3	5	62.5%	100.0%	100.0%	100.0%	100.0%	50.0%		

Figure S2. Overall patient survival after 1998 (after Tacrolimus)

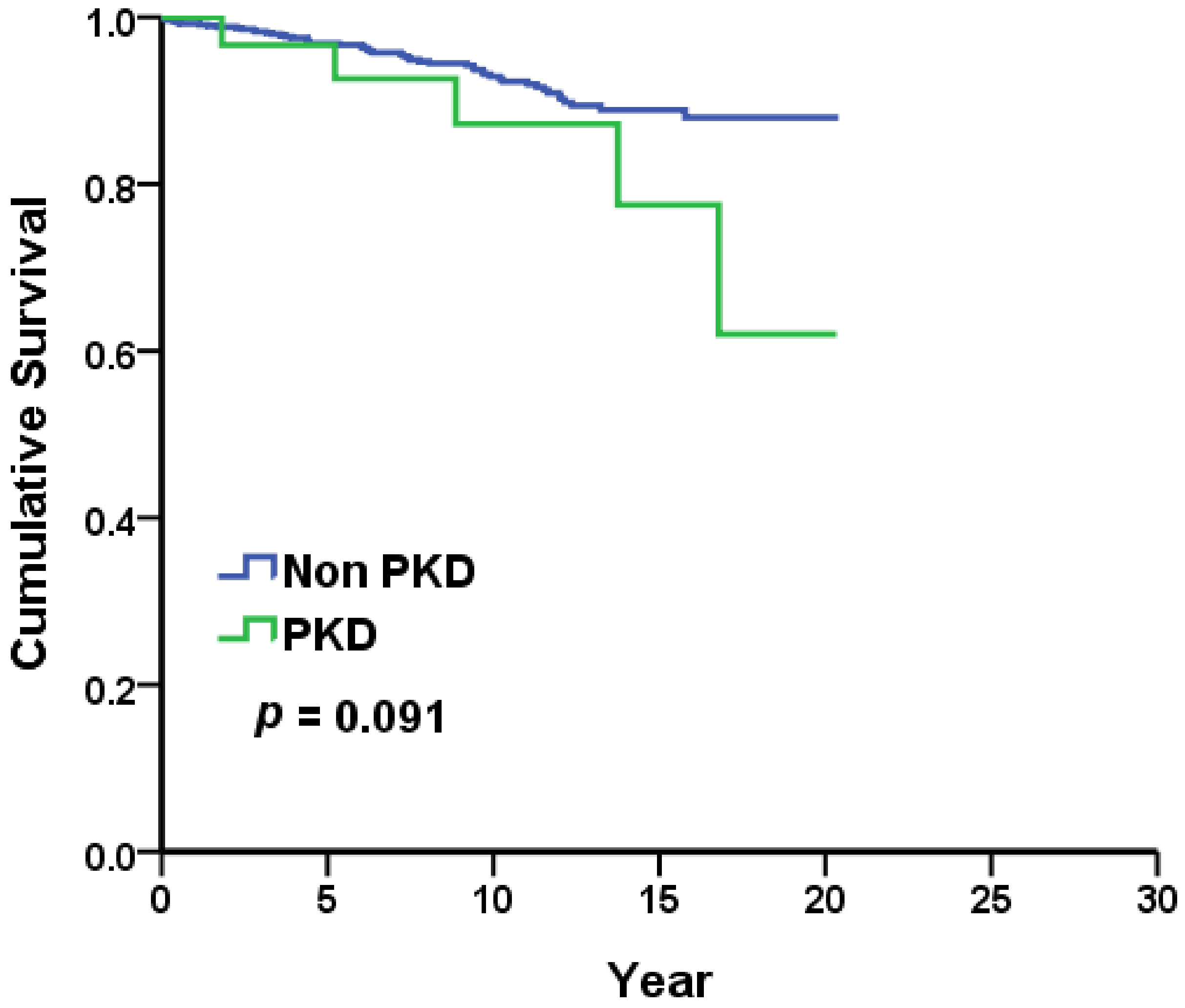

Overall survival (after 1998)

	Total	Death	Censored		Survival rate (%)						p for log rank
			n	%	5yr	10yr	15yr	20yr	25yr	30yr	
PKD											
No	803	52	751	93.5%	96.9%	92.9%	88.9%	88.9%			0.091
Yes	33	5	28	84.8%	96.7%	87.2%	77.5%	62.0%			

Table S1. Cause of death

Cause of death	ADPKD (n=8)	Non-ADPKD (n=85)
Cardiovascular disease	4 (50%)	40 (47.1%)
Cancer related	2 (25%)	13 (15.3%)
Infection	1 (12.5%)	22 (26%)
Others	1 (12.5%)	10 (11.8)

Table S2. Detailed information of types of malignancy

**Types of malignancy**	**ADPKD (n=18)**	**Non-ADPKD (n=360)**
Hematologic	0	17
Colon	4	78
Liver	2	78
Lung	4	50
Prostate	0	24
Bladder	4	23
Kidney	4	10
Others	0	80

Others in non-ADPKD: head and neck (n=10), esophagus (n=12), stomach (n=10), small intestine (n=1), pancreas (n=3), skin (n=11), female breast (n=11), uterus (n=14), brain (n=1), and thyroid (n=7).
